# IFN-α-Induced Upregulation of CCR5 Leads to Expanded HIV Tropism In Vivo

**DOI:** 10.1371/journal.ppat.1000766

**Published:** 2010-02-19

**Authors:** Cheryl A. Stoddart, Mary E. Keir, Joseph M. McCune

**Affiliations:** 1 Division of Experimental Medicine, Department of Medicine, San Francisco General Hospital, University of California, San Francisco, San Francisco, California, United States of America; 2 Gladstone Institute of Virology and Immunology, San Francisco, California, United States of America; 3 Biomedical Sciences Graduate Program, University of California, San Francisco, San Francisco, California, United States of America; University of Geneva, Switzerland

## Abstract

Chronic immune activation and inflammation (e.g., as manifest by production of type I interferons) are major determinants of disease progression in primate lentivirus infections. To investigate the impact of such activation on intrathymic T-cell production, we studied infection of the human thymus implants of SCID-hu Thy/Liv mice with X4 and R5 HIV. X4 HIV was observed to infect CD3^−^CD4^+^CD8^−^CXCR4^+^CCR5^−^ intrathymic T-cell progenitors (ITTP) and to abrogate thymopoiesis. R5 HIV, by contrast, first established a nonpathogenic infection of thymic macrophages and then, after many weeks, began to replicate in ITTP. We demonstrate here that the tropism of R5 HIV is expanded and pathogenicity enhanced by upregulation of CCR5 on these key T-cell progenitors. Such CCR5 induction was mediated by interferon-α (IFN-α) in both thymic organ cultures and in SCID-hu mice, and antibody neutralization of IFN-α in R5 HIV-infected SCID-hu mice inhibited both CCR5 upregulation and infection of the T-cell progenitors. These observations suggest a mechanism by which IFN-α production may paradoxically expand the tropism of R5 HIV and, in so doing, accelerate disease progression.

## Author Summary

Human immunodeficiency virus (HIV), a lentivirus, is the causative agent of AIDS. Chronic immune activation and inflammation are major determinants of disease progression in primate lentivirus infections and are associated with the production of type I interferon. To investigate the impact of type I interferon on HIV infection, we studied the human thymus implants of SCID-hu Thy/Liv mice infected with HIV that uses either CXCR4 (X4 HIV) or CCR5 (R5 HIV) as a coreceptor. X4 HIV was observed to infect T-cell progenitors in the thymus and to disrupt T-cell production by that organ. R5 HIV, by contrast, first established a nondisruptive infection of thymic macrophages and then began to infect intrathymic T-cell progenitors. We report here that the tropism of R5 HIV is expanded and T-cell disruption enhanced by increased expression of CCR5 on these key T-cell progenitors. Such CCR5 induction was mediated by interferon-α (IFN-α) in both thymic organ cultures and in SCID-hu mice. Moreover, antibody neutralization of IFN-α in R5 HIV-infected SCID-hu mice inhibited both CCR5 upregulation and infection of the T-cell progenitors. These observations suggest a mechanism by which IFN-α may paradoxically expand the tropism of R5 HIV and accelerate disease progression.

## Introduction

HIV disease progression is marked by chronic immune activation associated with accelerated destruction of T cells in the periphery and diminished production of new T cells from progenitors in the thymus and elsewhere [1],[2]. Although the detection of X4 HIV as the predominant viral species in peripheral blood is clearly associated with a higher risk of disease progression, about half of patients progress to AIDS in the presence of R5 viruses alone [3],[4] or with only the transient appearance of X4 virus [5]. Since it is just a small fraction of CD4^+^ target cells that express the CCR5 coreceptor [6], the mechanisms underlying such intrinsic R5 virus pathogenicity remain unclear. Given the association between high levels of T-cell activation and more rapid disease progression in untreated HIV-infected individuals [7], however, it is possible that such activation might induce the upregulation of CCR5 and expand the tropism of R5 HIV to include essential T-cell progenitors that are normally spared.

To address the hypothesis that R5 HIV infection might lead to such an indirect expansion of tropism in vivo, we investigated the course of R5 HIV infection in the SCID-hu Thy/Liv mouse model of human T-cell production. This small animal model, in which severe combined immunodeficient (C.B-17 SCID) mice are implanted with human fetal thymus and liver under the kidney capsule, supports multilineage human hematopoiesis, including T lymphopoiesis, for periods up to one year [8] and represents a venue in which to study the effects of HIV on human thymopoiesis in vivo. After inoculation with X4 HIV, a key population of ITTPs (CD3^−^CD4^+^CD8^−^CXCR4^+^CCR5^−^) is rapidly infected and destroyed, impeding thymocyte maturation and depleting the implants of thymocytes within 4–5 weeks [9],[10]. In contrast, rapid destruction of the thymic organ is not observed after infection with the R5 isolate Ba-L, which follows a biphasic process involving nonpathogenic replication in medullary stromal macrophages followed by cytopathic replication in thymocytes after 6 weeks of infection [11]. CCR5 is expressed at much lower levels than CXCR4 (<5% versus 30–40% of thymocytes) at all stages of T-cell development in the thymus [6],[12],[13], and this may explain the decreased pathogenicity of R5 HIV in that organ.

We demonstrate here that R5 HIV causes eventual depletion of thymocytes that is associated with *de novo* IFN-α-mediated upregulation of CCR5 on ITTP, rendering these key progenitor cells permissive for R5 HIV infection and depletion. Moreover, we show that monoclonal antibody (MAb) neutralization of IFN-α in SCID-hu Thy/Liv mice inhibits CCR5 induction after HIV infection and prevents infection of ITTP with R5 HIV. The observation that IFN-α may be a driving force behind expanded HIV tropism in vivo offers a proximal mechanism for the relationship between immune activation and disease progression and suggests that immunomodulatory agents that suppress the production or the effects of IFN-α may serve to slow disease progression in the HIV-infected host.

## Results

### HIV infection is associated with CCR5 induction and infection of thymocyte progenitors

**Figure 1:**
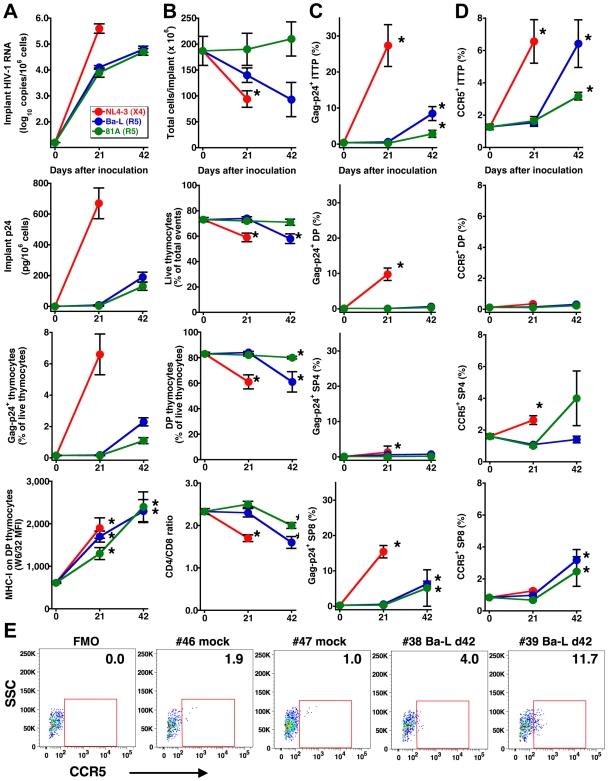
HIV infection is associated with increases in CCR5 expression and detection of R5 HIV in thymocyte progenitors. Increases in (A) viral load (as determined by implant viral RNA, p24, and Gag-p24^+^ thymocytes) and MHC-I expression, (B) thymocyte depletion (as determined by total cellularity, the percentage of viable thymocytes and of DP thymocytes, and the CD4/CD8 ratio), (C) Gag-p24^+^ thymocyte subpopulations and (D) CCR5 expression by thymocyte subpopulations in Thy/Liv implants with time after inoculation with 1,000 TCID_50_ HIV NL4-3, Ba-L, or 81A. Data are presented as mean±SEM for *n* = 7 or 8 mice per time point and virus, and asterisks indicate *P*<0.05 compared to mock-infected mice (day 0 data) by the Mann-Whitney U test. (E) CCR5 expression on ITTP from representative mock- and Ba-L-infected (day 42) implants. FMO is the fluorescence-minus one plot used for CCR5 gating, and percentages of CCR5^+^ ITTP are indicated. DP are “double-positive” CD3^int^CD4^+^CD8^+^ thymocytes, SP4 are “single-positive” CD3^+^CD4^+^CD8^–^ thymocytes, and SP8 are “single-positive” CD3^+^CD4^+^CD8^–^ thymocytes.

The human thymus implants of SCID-hu Thy/Liv mice were inoculated with the X4 HIV clone NL4-3, the R5 HIV isolate Ba-L, or a chimeric R5 clone of NL4-3 containing the V1-V3 *env* regions of Ba-L (81A) and monitored for viral replication and thymocyte depletion at 21 and 42 days. As expected from our previous work in the Thy/Liv model [11],[14],[15],[16], viral replication resulted in time-dependent increases in implant HIV RNA, p24, Gag-p24^+^ thymocytes, and MHC class I expression on CD3^int^CD4^+^CD8^+^ (double-positive, DP) thymocytes (Figure 1A). Viral replication was accompanied by time-dependent decreases in implant cellularity, thymocyte viability, percentage of DP thymocytes, and CD4/CD8 ratio (Figure 1B) that were more rapid and of greater magnitude for X4 than for R5 HIV, a finding consistent with the far greater of expression of CXCR4 than CCR5 on human thymocyte subpopulations [6].

The slow but evident pathogenicity of R5 HIV may be dependent upon inductive events that take place after infection. For instance, progressive sequence variation in the *env* gene may enable a “switch” of envelope glycoproteins to a pathogenic X4 phenotype. Alternatively, R5 viral pathogenesis may proceed in a time-dependent manner through infection of ITTP, which constitute a minor but key thymocyte progenitor subpopulation, in a manner analogous to X4 thymic pathogenesis [10]. Since we have previously found that an R5-to-X4 phenotypic switch is not detectable during thymic infection with Ba-L [11], we more closely evaluated the possibility that ITTP, which are normally CCR5-negative [6],[12], might be infected at some time point after virus inoculation.

Intracellular Gag-p24 staining in concert with surface staining for CD3, CD4, and CD8 revealed that ITTP were infected by Ba-L and 81A at day 42 but not day 21 (Figure 1C). This finding was unexpected because ITTP do not normally express CCR5 [6] and were thus not considered targets of R5 HIV infection, in marked contrast to the susceptibility of ITTP to X4 HIV infection as a consequence of high-level expression of CXCR4 [6]. Reasoning that CCR5 expression might be indirectly induced by HIV infection, we evaluated CCR5 expression on thymocyte subpopulations after HIV inoculation and found, at day 42 but not day 21, statistically significant increases in the percentage of CCR5-expressing ITTP [to 6.4±1.5% (*P* = 0.017) for Ba-L and to 3.2±0.2% for 81A (*P* = 0.001) versus a mean of 1.3±0.2% for mock-infected implants] (Figure 1D and E). Less dramatic, but still statistically significant increases in CCR5-positive CD3^+^CD4^−^CD8^+^ (single-positive, SP8) thymocytes were also observed, as has been reported previously in NOD/SCID-hu BLT mice infected intravaginally with HIV and attributed to a heightened state of immune activation [17]. Significant increases in the percentage of CCR5^+^ ITTP were also observed in X4 NL4-3-infected implants (Figure 1D). Treatment of SCID-hu Thy/Liv mice with 3TC (lamivudine) inhibited the induction of CCR5 on thymic progenitors and prevented Ba-L-mediated thymocyte depletion (data not shown). These results indicate that induction of CCR5 in HIV-infected Thy/Liv implants occurs in a time-dependent manner that is dependent on active HIV (R5 or X4) replication.

### CCR5 expression on ITTP is induced by IFN-α

**Figure 2:**
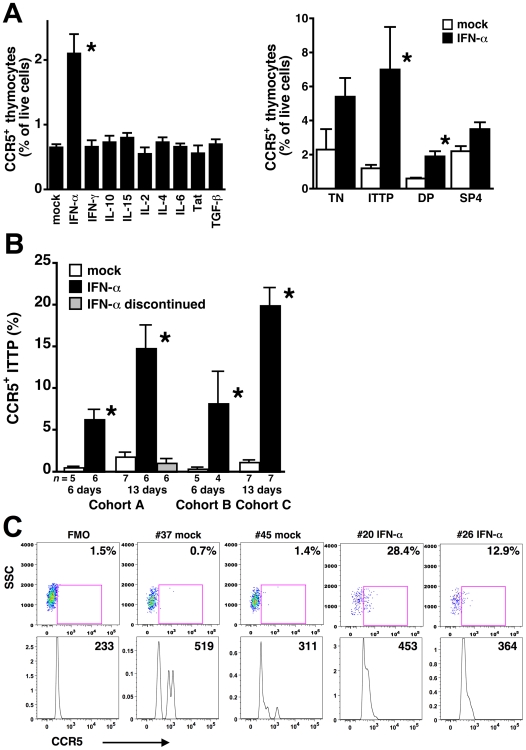
IFN-α induces CCR5 expression on thymocyte progenitors both in vitro and in vivo. (A) Thymus organ cultures were incubated in triplicate with the indicated cytokines or with Tat for 3 days, and dispersed cells were stained with MAbs against CD3, CD4, CD8, and CCR5 for flow cytometry. Only treatment with IFN-α significantly increased CCR5 expression on thymocytes (left), and the ITTP subset showed the greatest increase (right). Data are representative of three independent experiments. TN are “triple-negative” CD3^−^CD4^−^CD8^−^ thymocyte progenitors. (B) SCID-hu Thy/Liv mice from three cohorts (A, B, and C) were treated with IFN-α or sterile water (mock) by once-daily i.p. injection for 6 or 13 days, and Thy/Liv implants were collected and stained one day after the last injection or after 7 additional days of no treatment. IFN-α significantly increased the percentage of CCR5^+^ ITTP, which normalized within 7 days of treatment cessation. Asterisks indicate *P*<0.05 compared to mock-treated cultures or mice by the Mann-Whitney U test. (C) CCR5 expression on ITTP from representative mock- and IFN-α-treated (cohort C) mice. FMO is the fluorescence-minus one plot used for CCR5 gating, and percentages of CCR5^+^ ITTP are indicated. The mean fluorescence intensity of the CCR5^+^ ITTP is shown in the histogram below each dot plot.

Previous reports have demonstrated that CCR5 expression can be increased on several cell types after treatment with cytokines including IL-2 [18], IL-4 [19], IL-10 [20],[21], IL-15 [22], TGF-β [23], and IFN-γ [24], and with HIV Tat [25]. When human thymic organ cultures were incubated with these and other cytokines, significant induction of CCR5 expression on human thymocytes was only observed after treatment with IFN-α (Figure 2A). Analysis of CCR5 expression on thymocyte subpopulations demonstrated statistically significant upregulation on ITTP (Figure 2B), the same key subpopulation found to upregulate CCR5 in the HIV infected thymic implant and which we have previously reported to express the IFN-α/β receptor [26]. This receptor is expressed at high levels on ITTP and at progressively lower levels on more mature thymocytes (e.g., DP, SP4, and SP8 thymocytes) [26]. The ability of IFN-α to induce expression of CCR5 is consistent with the presence of STAT-binding sites at nt −55 and −116 in the CCR5 promoter; mutation of the proximal STAT site nearly abolishes promoter activity [27].

To determine whether IFN-α can induce expression of CCR5 on ITTP in vivo, we treated groups of SCID-hu Thy/Liv mice in three separate cohorts (A, B, and C) with IFN-α2b (Intron A or pegylated interferon alfa-2b) by once-daily intraperitoneal (i.p.) injection for 6 or 13 days. Significant increases in the percentage of CCR5^+^ ITTP were observed at both time points, normalizing to pretreatment levels after discontinuation of IFN-α (Figure 2B and C). Treatment with IFN-α for 13 days had no effect on the percentage and absolute number of ITTP or other more mature thymocyte subpopulations present in the implants (data not shown), so it is unlikely that the increase in CCR5^+^ cells is the result of either IFN-α-mediated apoptosis of CCR5-negative ITTP or an increased rate of CCR5^+^ ITTP cell division. Of note, the percentage of CCR5^+^ ITTP in IFN-α-treated mice (means in the three cohorts: 6–20%, range: 3–37%) (Figure 2B and C) tended to be higher than that found in Ba-L-infected mice at day 42 (mean: 6%, range: 0–12%) (Figure 1D and E), a difference that may be the result of virus-mediated depletion of infected CCR5^+^ progenitors.

### The presence of CCR5^+^ ITTP is highly correlated with viral load and thymocyte depletion after HIV infection

**Figure 3:**
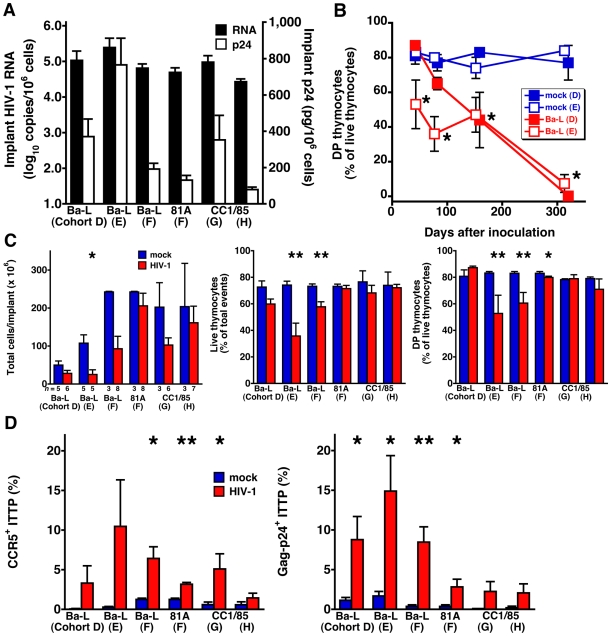
Thymocyte depletion and CCR5 induction on ITTP in six SCID-hu Thy/Liv mouse cohorts infected with R5 HIV Ba-L, 81A, or CC1/85. (A) Implant viral RNA and p24 42–49 days after inoculation. Data for cohort F (81A) are from Figure 1. (B) Time course of DP thymocyte depletion in Ba-L-infected cohorts D and E. (C) Implant cellularity, thymocyte viability, and percentage of DP thymocytes and (D) percentage of CCR5-expressing and Gag-p24^+^ ITTP for each of the five cohorts 42–49 days after inoculation. *P<0.05, **P<0.01 compared to mock-infected mice by the Mann-Whitney U test.

To examine more closely the relationship between CCR5^+^ induction on ITTP, thymic organ infection, and thymocyte depletion, we studied two additional SCID-hu Thy/Liv cohorts (D and E) inoculated with Ba-L plus two cohorts (G and H) inoculated with the R5 isolate, CC1/85. Data for these cohorts were analyzed together with the data obtained from the Ba-L and 81A-infected mice shown in Figure 1 (cohort F). Implant viral loads measured 42–49 days after inoculation were within 1.0 log_10_ across all six infected groups (means of 4.4–5.4 log_10_ copies HIV RNA and 80–800 pg p24 per 10^6^ cells) (Figure 3A). For the two additional SCID-hu cohorts inoculated with Ba-L (D and E), implants collected at much later time points (up to one year) after inoculation showed progressively more severe thymocyte depletion, while mock-infected implants remained intact with ∼80% DP thymocytes (Figure 3B). As we have reported previously [11], such depletion became noticeable 6 weeks after inoculation with Ba-L; we accordingly focused on implants collected from the five infected cohorts at this time (i.e., days 42–49) (Figure 3C). Although the decreases in implant cellularity, thymocyte viability, and percentage of DP thymocyte were often not statistically significant when individual experiments were compared (likely the result of the small number of mock-infected mice), animals in the R5 HIV-infected cohorts showed a trend towards decreases in each of these parameters. Concomitantly, there were increases in the percentage of CCR5^+^ ITTP compared to mock-infected mice, and the percentages of CCR5^+^ ITTP were comparable to the percentages of ITTP that were Gag-p24^+^ (Figure 3D).

**Figure 4:**
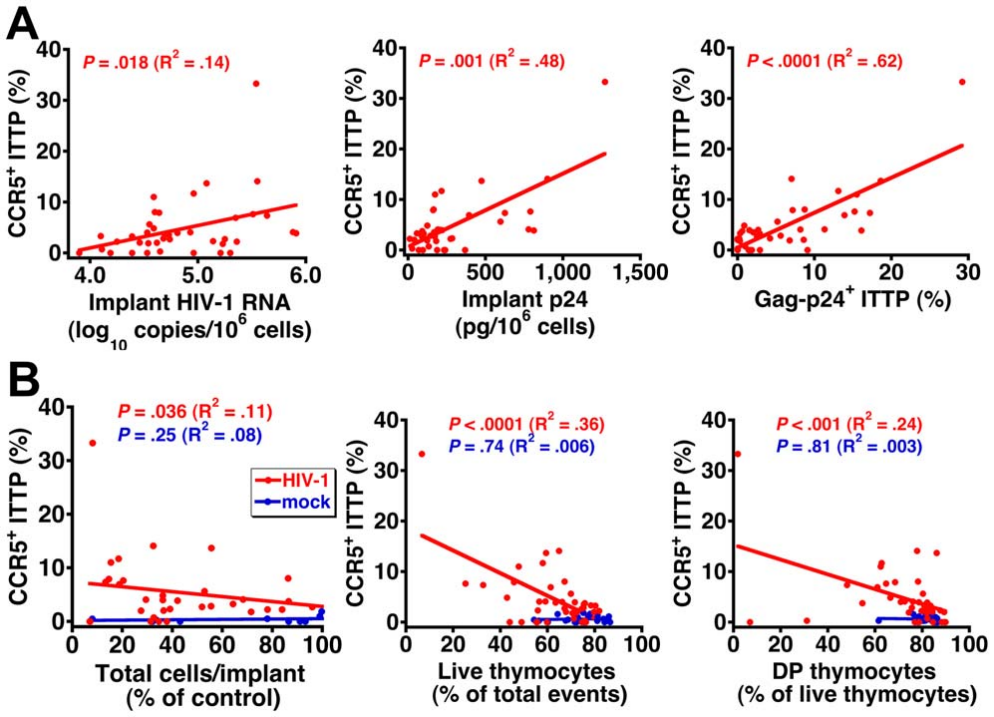
Correlation between CCR5 induction on ITTP, HIV replication, and thymocyte depletion. The percentage of CCR5-expressing ITTP is highly correlated with (A) viral load and (B) thymocyte depletion for each individual infected Thy/Liv implant in the six SCID-hu mouse cohorts shown in Figure 3. The percentage of control values for total cells per implant was calculated by dividing total cells per HIV-infected implant by the mean value for mock-infected implants from the same cohort to adjust for cohort-dependent variation in implant size.

To better document the relationship between CCR5 expression on ITTP and thymocyte depletion, data for each individual implant were plotted to show the correlation between the percentage of CCR5^+^ ITTP and viral load (Figure 4A) as well as the percentage of CCR5^+^ ITTP and thymocyte depletion (Figure 4B). Not only are the correlations highly significant for infected implants in statistical terms (e.g., *P*<0.0001 for CCR5^+^ ITTP versus both Gag-p24^+^ ITTP and thymocyte viability), but the proportion of CCR5^+^ ITTP corresponds closely to that of infected ITTP (Figure 4A). In contrast, there was no correlation between CCR5^+^ ITTP and markers of thymocyte depletion for mock-infected implants (Figure 4B). Accordingly, it is highly likely that induction of CCR5 expression on ITTP is a causal event precipitating thymocyte depletion after HIV infection.

### Neutralization of IFN-α in R5, but not X4, HIV-infected SCID-hu mice inhibits CCR5 upregulation and infection of ITTP

**Figure 5:**
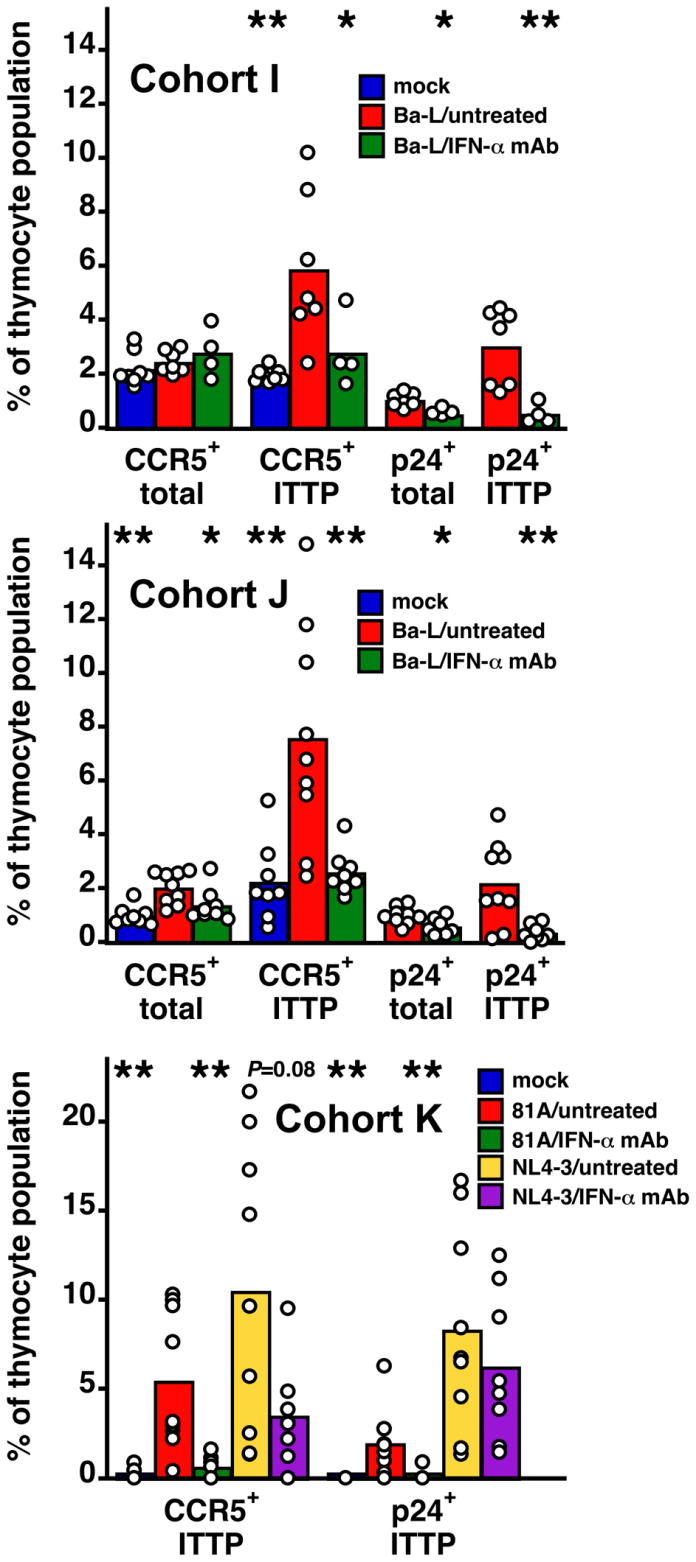
Neutralization of IFN-α inhibits CCR5 induction and HIV infection of ITTP after R5 HIV, but not after X4 HIV, inoculation. SCID-hu Thy/Liv mice infected (or not) with Ba-L (R5), 81A (R5), and NL4-3 (X4) were treated (or not) with IFN-α-neutralizing MAb (500 µg every other by i.p. injection) in three independent experiments. Columns represent means and open circles individual animals 43 days after Ba-L, 63 days after 81A, and 21 days after NL4-3 inoculation. *P<0.05, **P<0.01 compared to infected untreated mice by the Mann-Whitney U test.

To show definitively that upregulation of CCR5 on ITTP was mediated by IFN-α we treated three cohorts (I, J, and K) of SCID-hu Thy/Liv mice with a broadly neutralizing mouse MAb against multiple human IFN-α subtypes. Mice were treated by three times weekly i.p. injection, beginning 2 days before Ba-L, 81A, or NL4-3 inoculation and continuing until implant collection. For mice infected with Ba-L, neutralization of IFN-α was found to result in a lower percentage of CCR5^+^ ITTP (*P*<0.05 in cohort I and *P*<0.01 in cohort J), a lower percentage of Gag-p24^+^ total live thymocytes (*P*<0.05 in both cohort I and J), and a lower percentage of Gag-p24^+^ ITTP (*P*<0.01 in cohort I and J) (Figure 5). In cohort K, we directly compared the effects of IFN-α neutralization on HIV 81A (R5) and NL4-3 (X4) infection. This experiment was carried out with the expectation that infection of ITTP would be inhibited after 81A, but not after NL4-3, inoculation. We found that this was indeed the case: there was a 93% reduction (*P* = 0.005) in Gag-p24^+^ ITTP after IFN-α-treatment in 81A-infected mice yet an insignificant 25% reduction (*P* = 0.501) in Gag-p24^+^ ITTP in treated NL4-3-infected mice. This was accompanied by expected reductions in CCR5^+^ ITTP for both viruses (89% reduction for 81A; *P* = 0.004 and 67% reduction for NL4-3; *P* = 0.083). Given our previous data showing that infection of ITTP leads to interruption of thymopoiesis [10], these results indicate that IFN-α-induced upregulation of CCR5 on ITTP is likely to result in diminished production of T cells from the thymus.

## Discussion

R5 isolates of HIV have been associated with disease progression in HIV-infected individuals [28]. Likewise, as we have shown here, R5 HIV can be pathogenic in the SCID-hu Thy/Liv model of human thymopoiesis. Even though there is little CCR5 expression in the human thymus, R5 HIV was found to induce delayed but significant depletion of developing DP thymocytes and reduction in implant cellularity, and progression of R5 infection was found to correlate with the induction of CCR5 expression on early thymic progenitor cells. Such induction, in turn, is mediated by IFN-α both in vitro and in vivo. This finding is in contrast to a previous report showing that R5 HIV infection of thymic organ cultures induced CCR5 on CD4^+^ thymocytes through the production of IL-10 and TGF-β [29]. The ability of HIV to induce expression of its own coreceptor through the major antiviral cytokine, IFN-α likely evolved to dampen this antiviral defense mechanism, a counterbalancing act that has been likened to a détente through which virus and host achieve conditions for coexistence [30].

The above results indicate that expanded tropism of R5 HIV in the infected human thymus (to ITTP and DP thymocytes) is a secondary event that occurs after the induction of IFN-α production, most likely from plasmacytoid dendritic cells (pDC). These cells function as part of the innate immune response by secreting large quantities of IFN-α after contact or infection with a wide range of viruses, including HIV [31],[32],[33]. IL-3Rα^+^ pDC reside in the medulla of the human thymus [34], and we have previously shown that these cells produce IFN-α in response to HIV infection in both human thymic organ culture and in SCID-hu Thy/Liv mice [26]. Intrathymic pDC express both CXCR4 and CCR5 and are themselves targets for HIV replication [35], although it is not known if infection of these cells plays a role in IFN-α secretion. In sum, interactions between R5 HIV and pDC might lead indirectly to upregulation of CCR5 on cells that are normally not permissive for R5 infection. If so, these data point to a critical role for pDC-mediated IFN-α secretion in R5 HIV pathogenesis in the thymus of the SCID-hu mouse.

There is a low frequency of CCR5^+^ pDC in the CD3^−^CD4^+^CD8^−^ thymocyte population (unpublished observations), but we believe our results are due to upregulation of CCR5 on the T-lineage component of this population for the following reasons: First, in vitro IFN-α treatment results in upregulation of CCR5 on ITTP and DP (Figure 2A), cell populations that are T-lineage and that express high levels of the IFN-α/β receptor [26]. In vivo, the same phenomenon occurs (Figure 2B). Second, The fraction of CCR5^+^ ITTP is similar to the fraction of p24^+^ ITTP (Figure 3D), and there is a significant relationship between the two when analyzed in a large number of animals (Figure 4A). Finally, neutralizing anti-IFN-α antibody blocks the upregulation of CCR5 on the ITTP population (Figure 5). All of these data (especially the data in Figure 5) are most consistent with IFN-α induction of CCR5 on the CD3^−^CD4^+^CD8^−^CCR5^−^ ITTP and the CD3^+^CD4^+^CD8^+^CCR5^−^ DP populations, both of which are permissive for infection and replication of HIV.

These data also illustrate the importance of cell-cell interactions that can occur in lymphoid tissue after HIV infection with profound influence on the course of disease progression and that are not easily replicated in dispersed cell cultures. In addition, available in vitro culture systems do not persist for the periods of time required to measure the impact of these interactions on HIV pathogenesis. The observations in this study thus underscore the need for a closer evaluation of the dynamics of HIV infection within lymphoid organs and provide experimental justification for such tissue analysis within HIV-infected human subjects.

The finding that IFN-α can enhance HIV infectivity is surprising, especially given the potent antiviral activity against HIV we and others have reported in IFN-α-treated thymic organ cultures [26],[36]. These counterposing effects of IFN-α may occur simultaneously in pDC-containing tissue, thereby contributing to the slow progression of thymocyte depletion usually seen after R5 infection. Persistently high levels of IFN-α and of IFN-inducible genes are associated with more rapid disease progression in SIV-infected macaques [37],[38],[39]. In contrast, nonpathogenic SIV infections are associated with transient IFN-α responses, possibly due to the inability of the virus to activate pDC [40]. There is likely a complicated set of kinetics at play during HIV infection of the Thy/Liv implant in vivo, including but not limited to: the rate of viral replication and spread; the rate of induction of IFN-α in pDC; the rate of upregulation of CCR5 on thymocytes that express the IFN-α/β receptor; the rate at which these cells are infected and destroyed by R5 HIV; the rate at which they are replenished from earlier, CCR5^−^ progenitors; and, not least, the rate at which more mature DP thymocytes are depleted. We presume that the late events observed after HIV infection represent a sum total of these and other counterposing rates, resulting eventually in complete depletion of double-positive thymocytes (e.g., by day 300 in Figure 3B).

IFN-α has been shown to inhibit thymic T-cell differentiation in both the mouse [41] and human [42]. IFN-α-mediated inhibition of T-cell development may have also contributed to the depletion of thymocyte subsets observed in this study; however, we found that treatment of the mice with IFN-α for 13 days had no effect on the percentage and absolute number of ITTP or other more mature thymocyte subpopulations present in the Thy/Liv implants. It is possible that more prolonged exposure of the implants to IFN-α over months of HIV infection may have more deleterious cumulative effects on T-cell maturation than relatively short-term IFN-α treatment.

The role of chemokine coreceptor utilization in HIV disease progression has been studied extensively. The switch of viral phenotype from R5 to X4 has a profound and negative effect on absolute CD4 cell counts [43] and has been implicated as a determining factor in accelerated disease progression [5]. However, it appears that R5 HIV [28] and SIV [44] have the capacity to be pathogenic in their own right. HIV can also evolve in vivo with increased affinity for CCR5, thus acquiring the ability to infect cells expressing low levels of the coreceptor and potentially increasing pathogenicity [45],[46]. We present evidence here that CCR5 induction resulting from IFN-α secretion by pDC plays a significant role in the pathogenesis of R5 HIV in the human thymus implant of the SCID-hu Thy/Liv mouse. Given the close structural and functional similarities between this model and the intact human thymus [8] as well as prior evidence that HIV can infect the thymus in humans [47],[48],[49],[50],[51], it is likely that these observations are relevant not only to the HIV-infected child with abundant thymic tissue but also to the HIV-infected adult, in whom residual thymic function can continue to play a role in the de novo production of naïve T cells [1].

Since pDC are resident throughout the lymphoid system and migrate to inflamed lymph nodes [52], the expansion of R5 HIV tropism described here in the human thymus may also occur in other organs of the hematolymphoid system. Indeed, recent data indicate that IFN-α treatment causes significant increases in CCR5 mRNA expression in PBMC cultures from both HIV-infected and uninfected individuals [53],[54], and IFN-α treatment of patients with uveitis resulted in increases of CCR5 expression on peripheral blood CD4^+^ T cells [55]. Even if these events are restricted to thymic pDC, residual thymic function that persists in some adults with HIV disease [1] might thereby be abrogated. Alone or together, such interactions between HIV, pDC, and normally CCR5-negative target cells might underlie disease progression induced by R5 viruses in vivo.

## Materials and Methods

### Viruses

The following reagents were obtained through the AIDS Research and Reference Reagent Program, Division of AIDS, NIAID, NIH: pNL4-3 [56] from Dr. Malcolm Martin, HIV-1_Ba-L_ [57] from Dr. Suzanne Gartner, Dr. Mikulas Popovic and Dr. Robert Gallo, and p81A-4 [58] (Cat#11440) from Dr. Bruce Chesebro. CC1/85 [59] was generously provided by Drs. Shawn Kuhmann and John Moore. Ba-L is a low-passage isolate that has been propagated exclusively in human monocyte/macrophages [57], and CC1/85 is a well-characterized patient isolate that has also been minimally lab adapted [59],[60]. Working stocks of NL4-3 and 81A were prepared by lipofectamine (Invitrogen) transfection of 293T cells and collection of supernatants on day 2. Ba-L stock was generated in monocyte-derived macrophages with the supernatant collected on day 8, and CC1/85 stock was generated in phytohemagglutinin (PHA)-activated peripheral blood mononuclear cells (PBMC) with the supernatants collected on day 4. Virus stocks were titrated by limiting dilution for 50% tissue culture infectious doses (TCID_50_) in PHA-activated PBMC with p24 detection by ELISA on day 7 as previously described [61].

### HIV infection of SCID-hu Thy/Liv mice

Human fetal thymus and liver were obtained through services provided by a nonprofit organization (Advanced Bioscience Resources) in accordance with federal, state, and local regulations. Coimplantation of thymus and liver pieces under the kidney capsule to generate SCID-hu Thy/Liv mice and inoculation of the Thy/Liv implants with HIV was performed as described [14],[62]. Male C.B-17 SCID (model #CB17SC-M, homozygous, C.B-*Igh-1^b^*/IcrTac-*Prkdc^scid^*) mice were obtained at 6–8 weeks of age from Taconic, and cohorts of 50–60 SCID-hu Thy/Liv mice were implanted with tissues from a single donor. Implanted mice were maintained in a barrier facility under pathogen-free conditions and inoculated 18 weeks after implantation with 50 µl of stock virus (1,000 TCID_50_) or conditioned medium from PBMC cultures (mock infection) by direct injection into the implant. All procedures with mice were approved by the UCSF Institutional Animal Care and Use Committee. The Thy/Liv implants were collected from euthanized mice at the indicated time points, placed into sterile PBS-FBS, and dispersed through nylon mesh into a single cell suspension. Cells were counted and processed for p24 ELISA, branched DNA assay, and flow cytometry as previously described [14],[15].

### Flow cytometry

Dispersed implant cells were stained with MAbs against CD3, CD4, CD8, MHC class I, CCR5, and intracellular Gag-p24. Pellets containing 10^6^ cells were resuspended in 50 µl of a MAb mixture containing phycoerythrin cyanine dye CY7-conjugated anti-CD4 (BD Biosciences), phycoerythrin cyanine dye CY5.5-conjugated anti-CD8 (Caltag Laboratories), allophycocyanin cyanine dye CY7-conjugated anti-CD3 (eBiosciences), allophycocyanin-conjugated anti-CD195 (CCR5, clone 2D7) (BD Biosciences), and phycoerythrin-conjugated anti-W6/32 (DakoCytomation) in PBS containing 0.8 mg/ml human IgG (Biodesign International). Cells from one implant were also stained with conjugated, isotype-matched antibodies to control for nonspecific antibody binding. Cells were incubated for 30 min in the dark and washed two times with PBS/2% FBS. Cells were resuspended in 200 µl of a fixation/permeabilization mixture containing 1.25% human IgG (Biodesign International), 1.2% paraformaldehyde (Sigma), and 0.5% polyoxyethylenesorbitan (Tween 20, Sigma) in PBS/2% FBS. Cells were incubated for 60 min in the dark, washed two times with PBS/2% FBS, and then resuspended in 50 µl of PBS containing fluorescein isothiocyanate-conjugated anti-p24 (Beckman Coulter) and 0.8 mg/ml human IgG (Biodesign International). In addition, a “fluorescence minus one” (FMO) control was prepared in which the anti-p24-FITC was omitted from the antibody mixture to allow for discrimination of Gag-p24^+^ from Gag-p24^−^ cells. Cells were incubated for 30 min in the dark, washed twice with PBS/2% FBS, resuspended in 200 µl of PBS/2% FBS in 1.5-ml tubes, and analyzed on an LSR II (BD Biosciences) with FlowJo software (Tree Star). Optimization of fluorescence compensation for correction of fluorescence spectral overlaps emitted from the fluorescent conjugated antibodies was achieved by staining cells with each antibody alone plus anti-mouse Ig kappa chain and negative control BD CompBeads (BD Biosciences), as directed by the manufacturer. After collecting 100,000 total cell events, percentages of marker-positive (CD4^+^, CD8^+^, and DP) thymocytes in the implant samples were determined by first gating on a live lymphoid cell population identified by forward- and side-scatter characteristics and then by CD3 expression. In addition, the fraction of cells positive for Gag-p24 and CCR5 was determined for all thymocyte subpopulations in each implant (Figure S1). W6/32-positive mean fluorescence intensity (MFI) of DP thymocytes was determined for each sample, and CD4/CD8 ratios were calculated by dividing the percentage of CD4^+^ cells by the percentage of CD8^+^ cells for each individual implant.

### Treatment of SCID-hu Thy/Liv mice with IFN-α and IFN-α neutralizing MAb

SCID-hu Thy/Liv mice from three cohorts (A, B, and C) were treated with 10^6^ IU recombinant interferon alfa-2b (Schering), 10 µg pegylated interferon alfa-2b (Schering), or sterile water by once-daily i.p. injection for 6 or 13 days. Implants were collected and stained for flow cytometry either 1 or 7 days after the last IFN-α injection. SCID-hu Thy/Liv mice from three cohorts (I, J, and K) were treated with a mouse MAb with broadly neutralizing activity against multiple human IFN-αs (clone 9F3.18.5 [63], 500 µg every other day by i.p. injection) kindly provided by Drs. Andrew C. Chan and Kerstin Schmidt (Genentech) beginning 2 days before implant injection with Ba-L or 81A. The 9F3 MAb does not neutralize IFN-β [63].

### Cytokine treatment of human thymic organ cultures

Fetal thymus was dissected into small pieces and plated on sterile filters (Millipore) placed on gelatin sponges (Pharmacia and Upjohn) in 700 µl Yssel's medium containing 1% human serum (Gemini Bio-Products) in 24-well plates. Cultures were incubated in the presence of various cytokines or HIV Tat at concentrations shown previously to induce CCR5 upregulation, e.g., at 10 ng/ml for IL-10 [21], IL-15 [22], HIV Tat [25], and TGF-β [23]; 20 ng/ml for IL-4 [19] and IFN-γ [24]; and 20 IU/ml for IL-2 [18]. In the case of IFN-α, a dose of 1,000 IU/ml was selected on the basis of dose-ranging experiments, although CCR5 upregulation was observed at lower (300 IU/ml) IFN-α concentrations (data not shown). Cytokine-treated thymus cultures were dispersed after 3 days, and cells were stained with MAbs to CD3, CD4, CD8, and CCR5 for flow cytometry as described above.

### Statistical analysis

Results are expressed as means±SEM. Nonparametric statistical analysis was performed by use of the Mann-Whitney U test (StatView 5.0, Abacus Concepts), and correlation *P* values were generated by the correlation Z test (StatView).

## Supporting Information

Figure S1 Gating of thymocyte subpopulations and determination of CCR5^+^ and Gag-p24^+^ thymocytes in Thy/Liv implants. After collecting 100,000 total cell events, percentages of marker-positive (CD4^+^, CD8^+^, and DP) thymocytes in the implant samples were determined by first gating on a live lymphoid cell population identified by forward- and side-scatter characteristics and then by CD3 expression. In addition, the fraction of cells positive for Gag-p24 and CCR5 was determined for all thymocyte subpopulations in each implant. W6/32-positive mean fluorescence intensity (MFI) of DP thymocytes was determined for each sample, and CD4/CD8 ratios were calculated by dividing the percentage of CD4^+^ cells by the percentage of CD8^+^ cells for each individual implant. Data shown is for HIV Ba-L-infected SCID-hu mouse #39 (Figure 1E).
(0.90 MB PDF)

## References

[1] McCune JM (2001). The dynamics of CD4+ T-cell depletion in HIV disease.. Nature.

[2] Picker LJ (2006). Immunopathogenesis of acute AIDS virus infection.. Curr Opin Immunol.

[3] Schuitemaker H, Koot M, Kootstra NA, Dercksen MW, de Goede RE (1992). Biological phenotype of human immunodeficiency virus type 1 clones at different stages of infection: progression of disease is associated with a shift from monocytotropic to T-cell-tropic virus population.. J Virol.

[4] Tersmette M, Gruters RA, de Wolf F, de Goede RE, Lange JM (1989). Evidence for a role of virulent human immunodeficiency virus (HIV) variants in the pathogenesis of acquired immunodeficiency syndrome: studies on sequential HIV isolates.. J Virol.

[5] Shankarappa R, Margolick JB, Gange SJ, Rodrigo AG, Upchurch D (1999). Consistent viral evolutionary changes associated with the progression of human immunodeficiency virus type 1 infection.. J Virol.

[6] Berkowitz RD, Beckerman KP, Schall TJ, McCune JM (1998). CXCR4 and CCR5 expression delineates targets for HIV-1 disruption of T cell differentiation.. J Immunol.

[7] Hunt PW (2007). Role of immune activation in HIV pathogenesis.. Curr HIV/AIDS Rep.

[8] McCune JM (1996). Development and applications of the SCID-hu mouse model.. Semin Immunol.

[9] Jenkins M, Hanley MB, Moreno MB, Wieder E, McCune JM (1998). Human immunodeficiency virus-1 infection interrupts thymopoiesis and multilineage hematopoiesis in vivo.. Blood.

[10] Su L, Kaneshima H, Bonyhadi M, Salimi S, Kraft D (1995). HIV-1-induced thymocyte depletion is associated with indirect cytopathogenicity and infection of progenitor cells in vivo.. Immunity.

[11] Berkowitz RD, Alexander S, Bare C, Linquist-Stepps V, Bogan M (1998). CCR5- and CXCR4-utilizing strains of human immunodeficiency virus type 1 exhibit differential tropism and pathogenesis in vivo.. J Virol.

[12] Kitchen SG, Zack JA (1999). Distribution of the human immunodeficiency virus coreceptors CXCR4 and CCR5 in fetal lymphoid organs: implications for pathogenesis in utero.. AIDS Res Hum Retroviruses.

[13] Zamarchi R, Allavena P, Borsetti A, Stievano L, Tosello V (2002). Expression and functional activity of CXCR-4 and CCR-5 chemokine receptors in human thymocytes.. Clin Exp Immunol.

[14] Rabin L, Hincenbergs M, Moreno MB, Warren S, Linquist V (1996). Use of standardized SCID-hu Thy/Liv mouse model for preclinical efficacy testing of anti-human immunodeficiency virus type 1 compounds.. Antimicrob Agents Chemother.

[15] Stoddart CA, Bales CA, Bare JC, Chkhenkeli G, Galkina SA (2007). Validation of the SCID-hu Thy/Liv mouse model with four classes of licensed antiretrovirals.. PLoS ONE.

[16] Berkowitz RD, Alexander S, McCune JM (2000). Causal relationships between HIV-1 coreceptor utilization, tropism, and pathogenesis in human thymus.. AIDS Res Hum Retroviruses.

[17] Denton PW, Estes JD, Sun Z, Othieno FA, Wei BL (2008). Antiretroviral pre-exposure prophylaxis prevents vaginal transmission of HIV-1 in humanized BLT mice.. PLoS Med.

[18] Weissman D, Dybul M, Daucher MB, Davey RT, Walker RE (2000). Interleukin-2 up-regulates expression of the human immunodeficiency virus fusion coreceptor CCR5 by CD4+ lymphocytes in vivo.. J Infect Dis.

[19] Pedroza-Martins L, Gurney KB, Torbett BE, Uittenbogaart CH (1998). Differential tropism and replication kinetics of human immunodeficiency virus type 1 isolates in thymocytes: coreceptor expression allows viral entry, but productive infection of distinct subsets is determined at the postentry level.. J Virol.

[20] Juffermans NP, Paxton WA, Dekkers PE, Verbon A, de Jonge E (2000). Up-regulation of HIV coreceptors CXCR4 and CCR5 on CD4(+) T cells during human endotoxemia and after stimulation with (myco)bacterial antigens: the role of cytokines.. Blood.

[21] Sozzani S, Ghezzi S, Iannolo G, Luini W, Borsatti A (1998). Interleukin 10 increases CCR5 expression and HIV infection in human monocytes.. J Exp Med.

[22] Perera LP, Goldman CK, Waldmann TA (1999). IL-15 induces the expression of chemokines and their receptors in T lymphocytes.. J Immunol.

[23] Sato K, Kawasaki H, Nagayama H, Enomoto M, Morimoto C (2000). TGF-beta 1 reciprocally controls chemotaxis of human peripheral blood monocyte-derived dendritic cells via chemokine receptors.. J Immunol.

[24] Hariharan D, Douglas SD, Lee B, Lai JP, Campbell DE (1999). Interferon-gamma upregulates CCR5 expression in cord and adult blood mononuclear phagocytes.. Blood.

[25] Weiss JM, Nath A, Major EO, Berman JW (1999). HIV-1 Tat induces monocyte chemoattractant protein-1-mediated monocyte transmigration across a model of the human blood-brain barrier and up-regulates CCR5 expression on human monocytes.. J Immunol.

[26] Keir ME, Stoddart CA, Linquist-Stepps V, Moreno ME, McCune JM (2002). IFN-alpha secretion by type 2 predendritic cells up-regulates MHC class I in the HIV-1-infected thymus.. J Immunol.

[27] Liu R, Zhao X, Gurney TA, Landau NR (1998). Functional analysis of the proximal CCR5 promoter.. AIDS Res Hum Retroviruses.

[28] de Roda Husman AM, van Rij RP, Blaak H, Broersen S, Schuitemaker H (1999). Adaptation to promiscuous usage of chemokine receptors is not a prerequisite for human immunodeficiency virus type 1 disease progression.. J Infect Dis.

[29] Choudhary SK, Choudhary NR, Kimbrell KC, Colasanti J, Ziogas A (2005). R5 human immunodeficiency virus type 1 infection of fetal thymic organ culture induces cytokine and CCR5 expression.. J Virol.

[30] Garcia-Sastre A, Biron CA (2006). Type 1 interferons and the virus-host relationship: a lesson in detente.. Science.

[31] Fitzgerald-Bocarsly P (1993). Human natural interferon-alpha producing cells.. Pharmacol Ther.

[32] Patterson S, Rae A, Hockey N, Gilmour J, Gotch F (2001). Plasmacytoid dendritic cells are highly susceptible to human immunodeficiency virus type 1 infection and release infectious virus.. J Virol.

[33] Siegal FP, Kadowaki N, Shodell M, Fitzgerald-Bocarsly PA, Shah K (1999). The nature of the principal type 1 interferon-producing cells in human blood.. Science.

[34] Res P, Spits H (1999). Developmental stages in the human thymus.. Semin Immunol.

[35] Fong L, Mengozzi M, Abbey NW, Herndier BG, Engleman EG (2002). Productive infection of plasmacytoid dendritic cells with human immunodeficiency virus type 1 is triggered by CD40 ligation.. J Virol.

[36] Gurney KB, Colantonio AD, Blom B, Spits H, Uittenbogaart CH (2004). Endogenous IFN-alpha production by plasmacytoid dendritic cells exerts an antiviral effect on thymic HIV-1 infection.. J Immunol.

[37] Abel K, Rocke DM, Chohan B, Fritts L, Miller CJ (2005). Temporal and anatomic relationship between virus replication and cytokine gene expression after vaginal simian immunodeficiency virus infection.. J Virol.

[38] Brown KN, Wijewardana V, Liu X, Barratt-Boyes SM (2009). Rapid influx and death of plasmacytoid dendritic cells in lymph nodes mediate depletion in acute simian immunodeficiency virus infection.. PLoS Pathog.

[39] Lederer S, Favre D, Walters KA, Proll S, Kanwar B (2009). Transcriptional profiling in pathogenic and non-pathogenic SIV infections reveals significant distinctions in kinetics and tissue compartmentalization.. PLoS Pathog.

[40] Mandl JN, Barry AP, Vanderford TH, Kozyr N, Chavan R (2008). Divergent TLR7 and TLR9 signaling and type I interferon production distinguish pathogenic and nonpathogenic AIDS virus infections.. Nat Med.

[41] Lin Q, Dong C, Cooper MD (1998). Impairment of T and B cell development by treatment with a type I interferon.. J Exp Med.

[42] Schmidlin H, Dontje W, Groot F, Ligthart SJ, Colantonio AD (2006). Stimulated plasmacytoid dendritic cells impair human T-cell development.. Blood.

[43] Koot M, Keet IP, Vos AH, de Goede RE, Roos MT (1993). Prognostic value of HIV-1 syncytium-inducing phenotype for rate of CD4+ cell depletion and progression to AIDS.. Ann Intern Med.

[44] Kimata JT, Kuller L, Anderson DB, Dailey P, Overbaugh J (1999). Emerging cytopathic and antigenic simian immunodeficiency virus variants influence AIDS progression.. Nat Med.

[45] Gorry PR, Taylor J, Holm GH, Mehle A, Morgan T (2002). Increased CCR5 affinity and reduced CCR5/CD4 dependence of a neurovirulent primary human immunodeficiency virus type 1 isolate.. J Virol.

[46] Moore JP, Doms RW (2003). The entry of entry inhibitors: a fusion of science and medicine.. Proc Natl Acad Sci U S A.

[47] Davis AE (1984). The histopathological changes in the thymus gland in the acquired immune deficiency syndrome.. Ann N Y Acad Sci.

[48] Elie R, Laroche AC, Arnoux E, Guerin JM, Pierre G (1983). Thymic dysplasia in acquired immunodeficiency syndrome.. N Engl J Med.

[49] Grody WW, Fligiel S, Naeim F (1985). Thymus involution in the acquired immunodeficiency syndrome.. Am J Clin Pathol.

[50] Joshi VV, Oleske JM (1985). Pathologic appraisal of the thymus gland in acquired immunodeficiency syndrome in children. A study of four cases and a review of the literature.. Arch Pathol Lab Med.

[51] Joshi VV, Oleske JM, Saad S, Gadol C, Connor E (1986). Thymus biopsy in children with acquired immunodeficiency syndrome.. Arch Pathol Lab Med.

[52] Cella M, Jarrossay D, Facchetti F, Alebardi O, Nakajima H (1999). Plasmacytoid monocytes migrate to inflamed lymph nodes and produce large amounts of type I interferon.. Nat Med.

[53] Stylianou E, Yndestad A, Sikkeland LI, Bjerkeli V, Damas JK (2002). Effects of interferon-alpha on gene expression of chemokines and members of the tumour necrosis factor superfamily in HIV-infected patients.. Clin Exp Immunol.

[54] Serra C, Biolchini A, Mei A, Kotenko S, Dolei A (2008). Type III and I Interferons Increase HIV Uptake and Replication in Human Cells That Overexpress CD4, CCR5, and CXCR4.. AIDS Res Hum Retroviruses.

[55] Plskova J, Greiner K, Muckersie E, Duncan L, Forrester JV (2006). Interferon-alpha: a key factor in autoimmune disease?. Invest Ophthalmol Vis Sci.

[56] Adachi A, Gendelman HE, Koenig S, Folks T, Willey R (1986). Production of acquired immunodeficiency syndrome-associated retrovirus in human and nonhuman cells transfected with an infectious molecular clone.. J Virol.

[57] Gartner S, Markovits P, Markovitz DM, Kaplan MH, Gallo RC (1986). The role of mononuclear phagocytes in HTLV-III/LAV infection.. Science.

[58] Toohey K, Wehrly K, Nishio J, Perryman S, Chesebro B (1995). Human immunodeficiency virus envelope V1 and V2 regions influence replication efficiency in macrophages by affecting virus spread.. Virology.

[59] Connor RI, Sheridan KE, Ceradini D, Choe S, Landau NR (1997). Change in coreceptor use coreceptor use correlates with disease progression in HIV-1–infected individuals.. J Exp Med.

[60] Trkola A, Kuhmann SE, Strizki JM, Maxwell E, Ketas T (2002). HIV-1 escape from a small molecule, CCR5-specific entry inhibitor does not involve CXCR4 use.. Proc Natl Acad Sci U S A.

[61] Stoddart CA, Liegler TJ, Mammano F, Linquist-Stepps VD, Hayden MS (2001). Impaired replication of protease inhibitor-resistant HIV-1 in human thymus.. Nat Med.

[62] Namikawa R, Weilbaecher KN, Kaneshima H, Yee EJ, McCune JM (1990). Long-term human hematopoiesis in the SCID-hu mouse.. J Exp Med.

[63] Chuntharapai A, Lai J, Huang X, Gibbs V, Kim KJ (2001). Characterization and humanization of a monoclonal antibody that neutralizes human leukocyte interferon: a candidate therapeutic for IDDM and SLE.. Cytokine.

