# Supplementary material for: IFN-α-Induced Upregulation of CCR5 Leads to Expanded HIV Tropism In Vivo
Source: PLoS Pathog. 2010 Feb 19;6(2):e1000766. doi: 10.1371/journal.ppat.1000766 (PMC2824759; doi:10.1371/journal.ppat.1000766)

Supplemental Figure 1. Gating of thymocyte subpopulations and determination of $CCR5^+$ and Gag-p24$^+$ thymocytes in Thy/Liv implants. After collecting 100,000 total cell events, percentages of marker-positive ($CD4^+$, $CD8^+$, and DP) thymocytes in the implant samples were determined by first gating on a live lymphoid cell population identified by forward- and side-scatter characteristics and then by CD3 expression. In addition, the fraction of cells positive for Gag-p24 and CCR5 was determined for all thymocyte subpopulations in each implant. W6/32-positive mean fluorescence intensity (MFI) of DP thymocytes was determined for each sample, and CD4/CD8 ratios were calculated by dividing the percentage of $CD4^+$ cells by the percentage of $CD8^+$ cells for each individual implant. Data shown is for HIV Ba-L-infected SCID-hu mouse #39 (Figure 1E).

EXPERIMENT: VQC-0701 TERMINATION DATE: 03/20/07

GROUP E

MOUSE_039

VIRUS: Ba-L TL WS2 D12

DRUG: None DOSAGE: 0 ug/dose

DOSAGE FREQUENCY: N/A

DRUG ROUTE: N/A

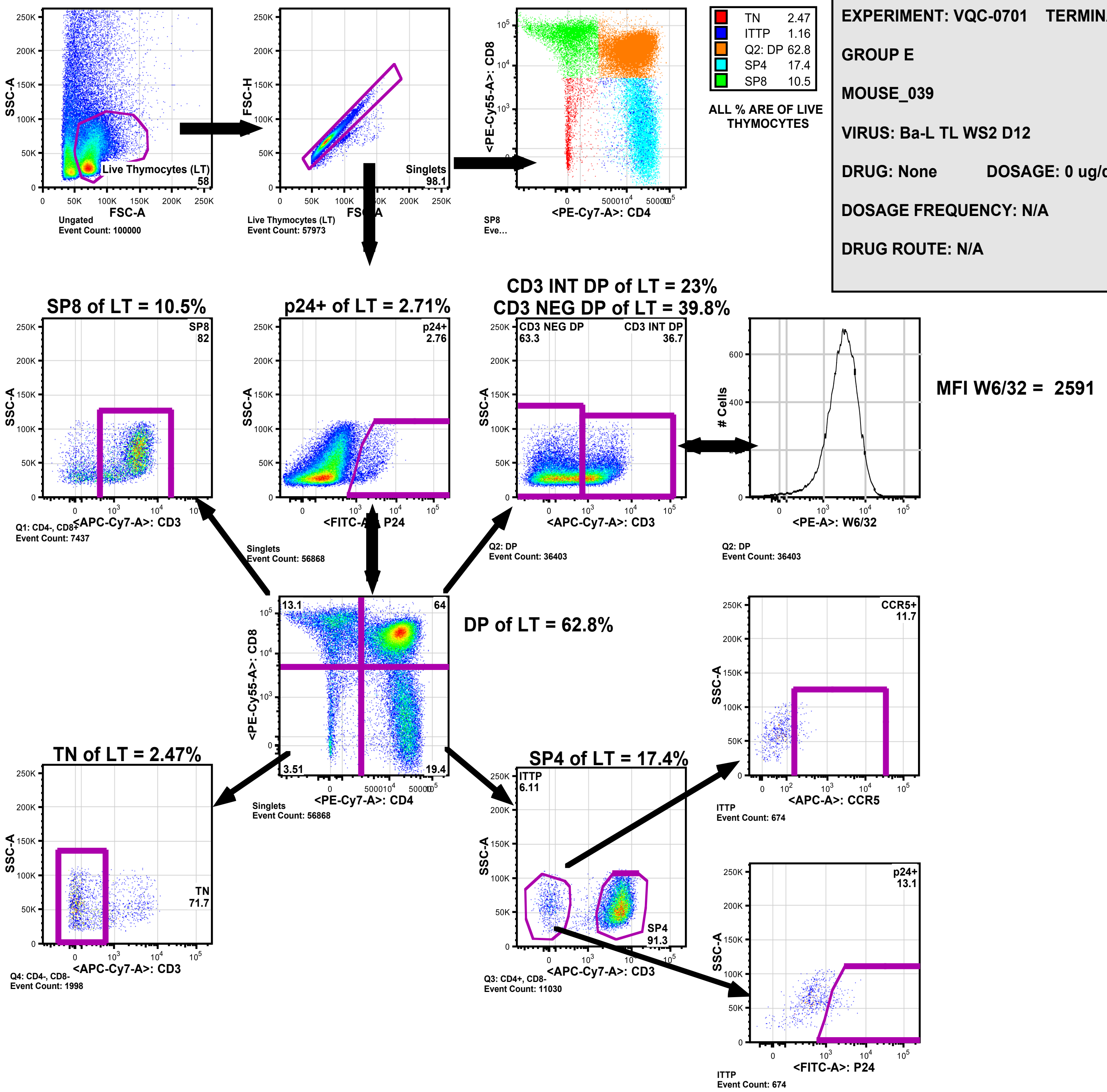

Supplement: Figure S1 — Gating of thymocyte subpopulations and determination of CCR5+ and Gag-p24+ thymocytes in Thy/Liv implants. After collecting 100,000 total cell events, percentages of marker-positive (CD4+, CD8+, and DP) thymocytes in the implant samples were determined by first gating on a live lymphoid cell population identified by forward- and side-scatter characteristics and then by CD3 expression. In addition, the fraction of cells positive for Gag-p24 and CCR5 was determined for all thymocyte subpopulations in each implant. W6/32-positive mean fluorescence intensity (MFI) of DP thymocytes was determined for each sample, and CD4/CD8 ratios were calculated by dividing the percentage of CD4+ cells by the percentage of CD8+ cells for each individual implant. Data shown is for HIV Ba-L-infected SCID-hu mouse #39 (Figure 1E). (0.90 MB PDF) [file ppat.1000766.s001.pdf]
